# Supplementary material for: Morphological and genetic variability in cosmopolitan tardigrade species—Paramacrobiotus fairbanksi Schill, Förster, Dandekar & Wolf, 2010
Source: Sci Rep. 2023 Oct 17;13:17672. doi: 10.1038/s41598-023-42653-6 (PMC10582252; doi:10.1038/s41598-023-42653-6)
Supplement: Supplementary file 13 — Supplementary Information 13. [file 41598_2023_42653_MOESM13_ESM.pdf]

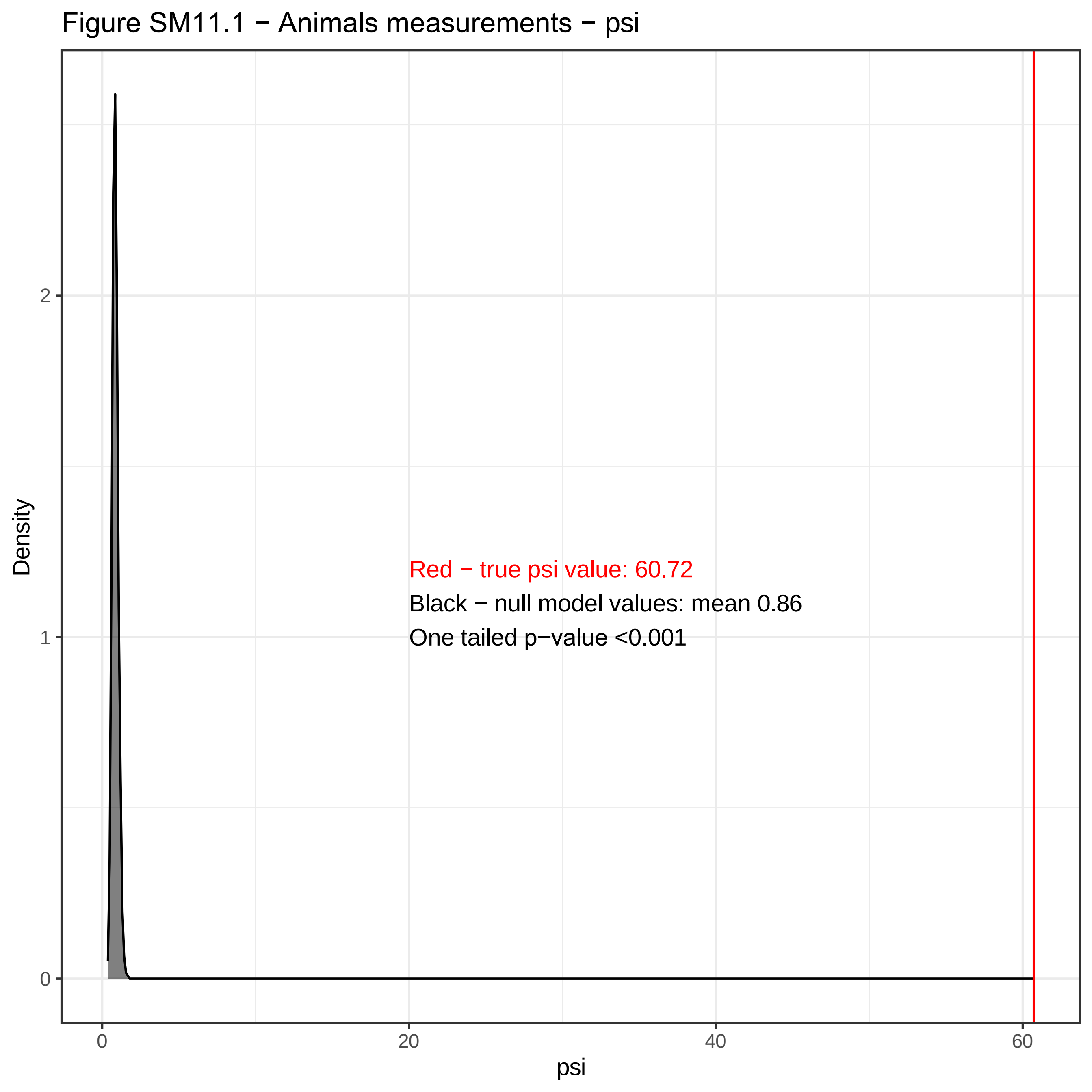
Figure SM11.1 − Animals measurements − psi
Red − true psi value: 60.72
Black − null model values: mean 0.86
One tailed p−value <0.001
Density
psi
0
1
2
0
20
40
60

Figure SM11.2 − Animals measurements − phi

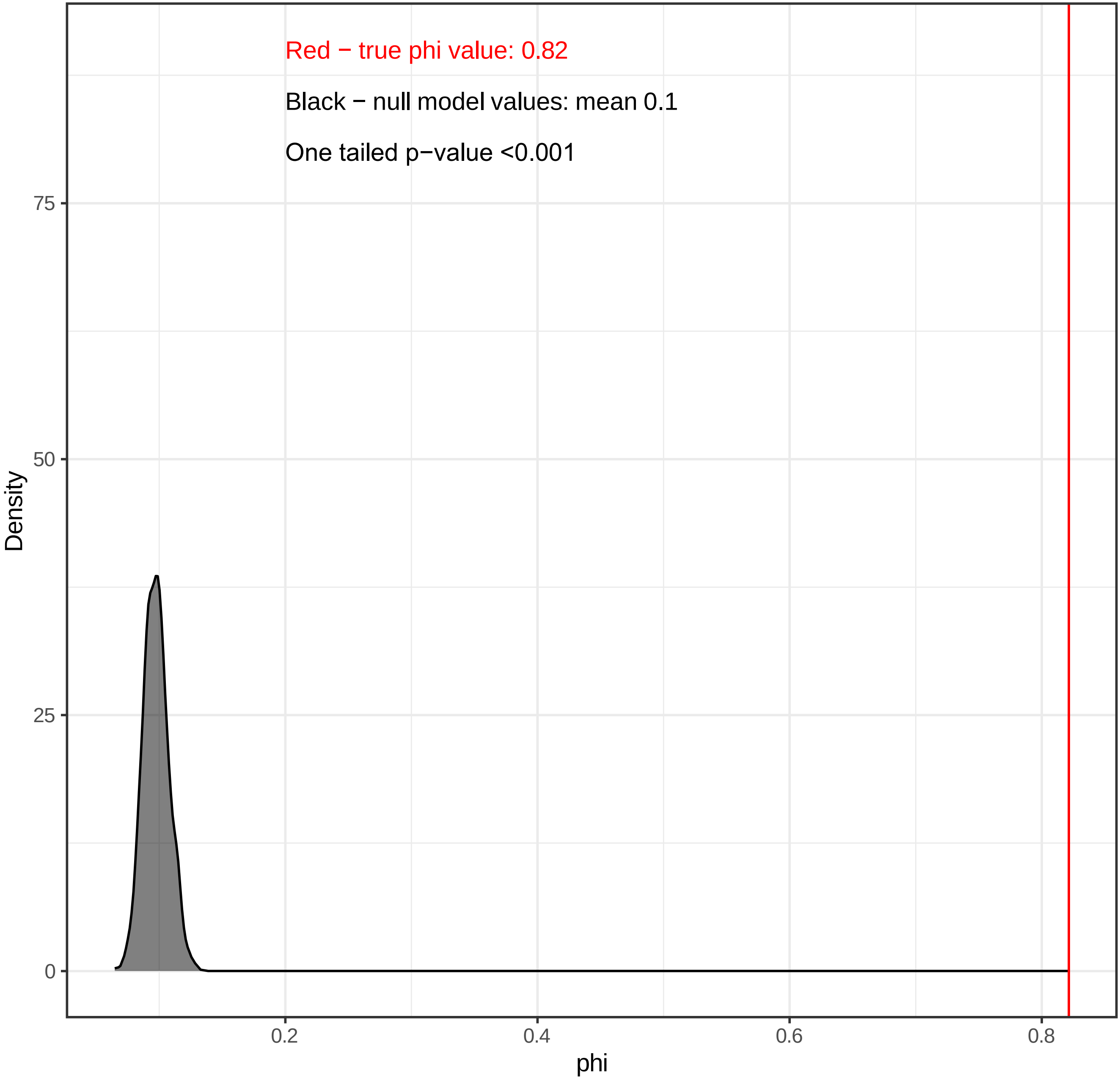

# Figure SM11.3 − Animals measurements − explained variance by component

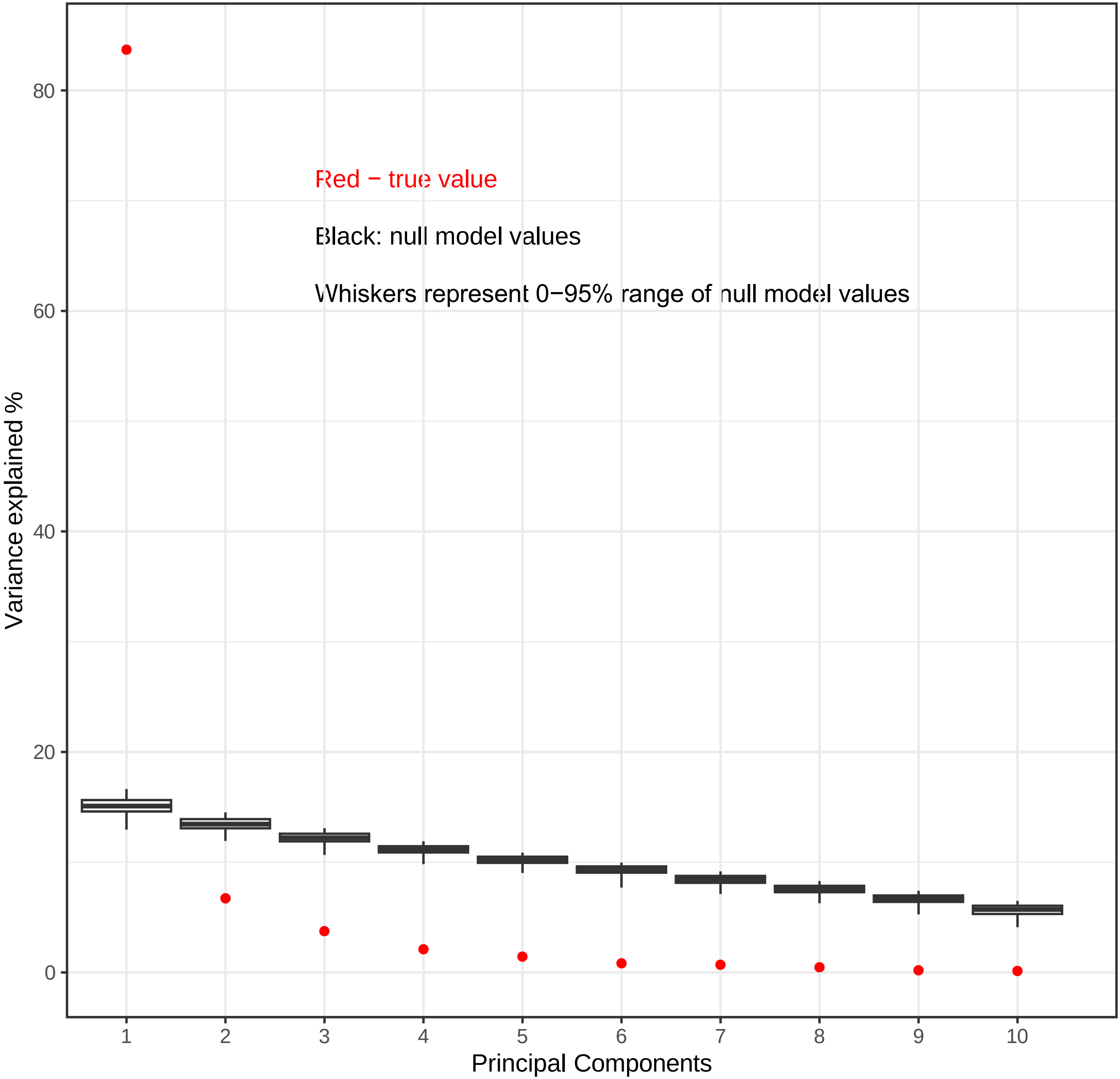

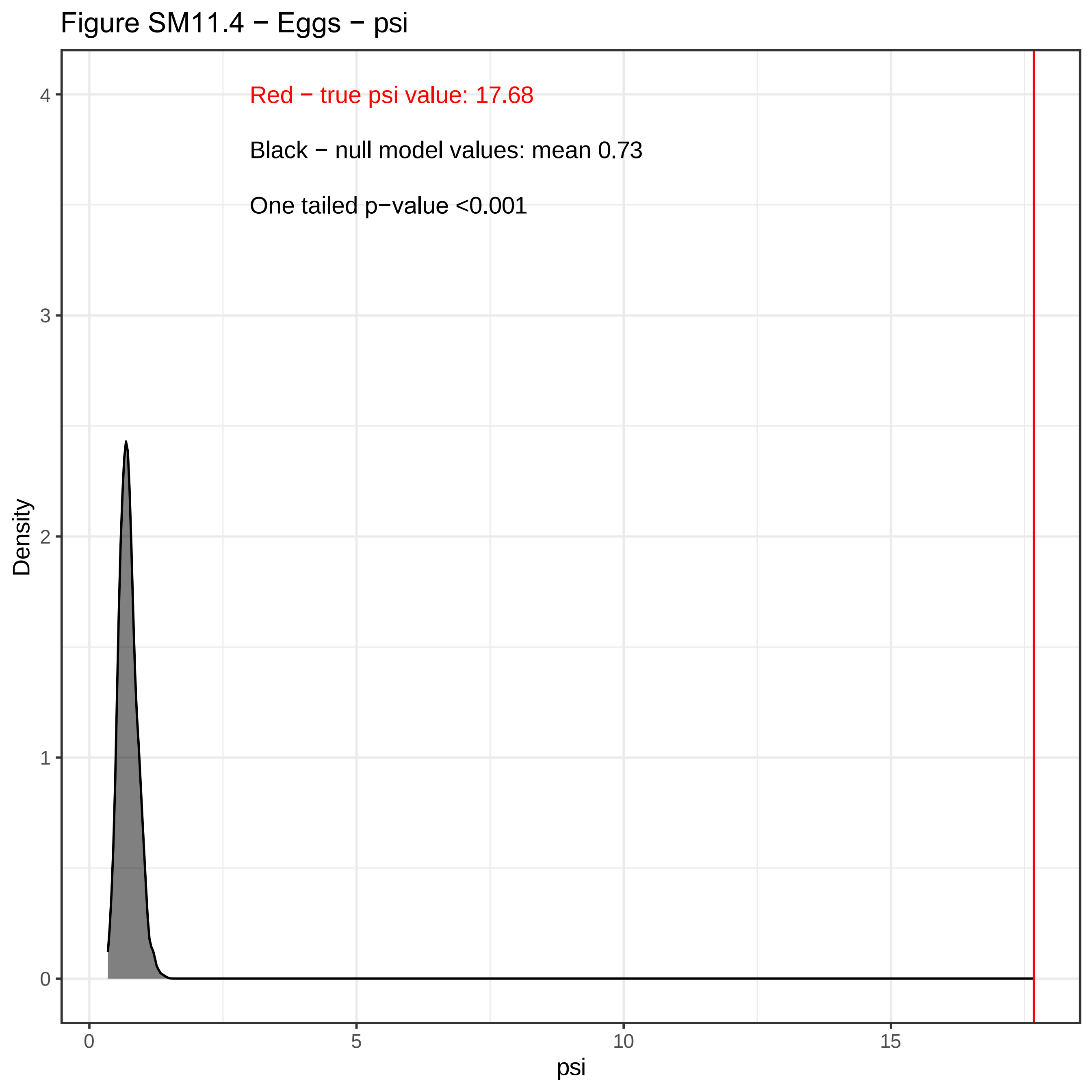
Figure SM11.4 − Eggs − psi
Red − true psi value: 17.68
Black − null model values: mean 0.73
One tailed p−value <0.001
Density
psi
0
1
2
3
4
0
5
10
15

Figure SM11.5 − Eggs − phi

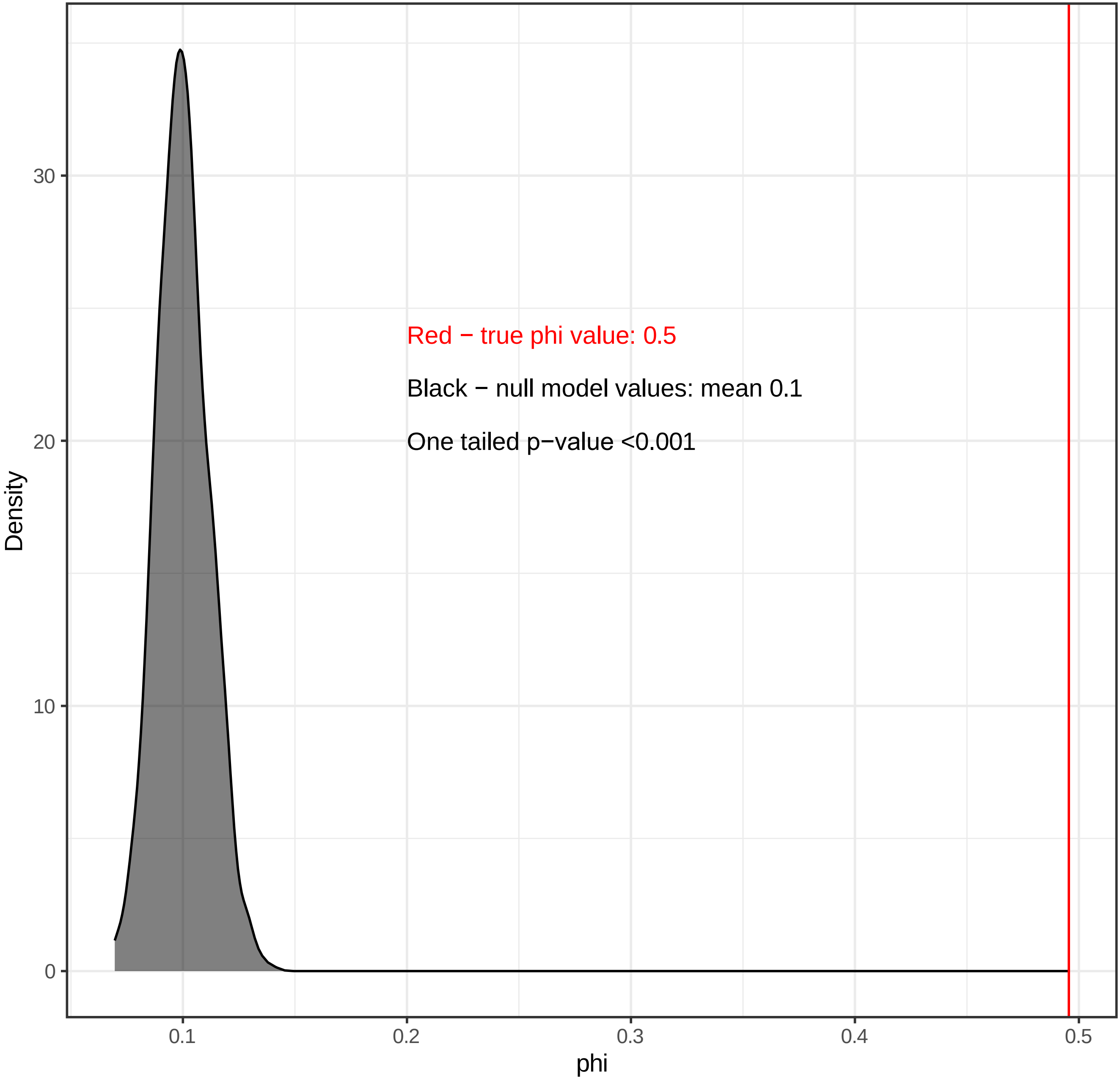

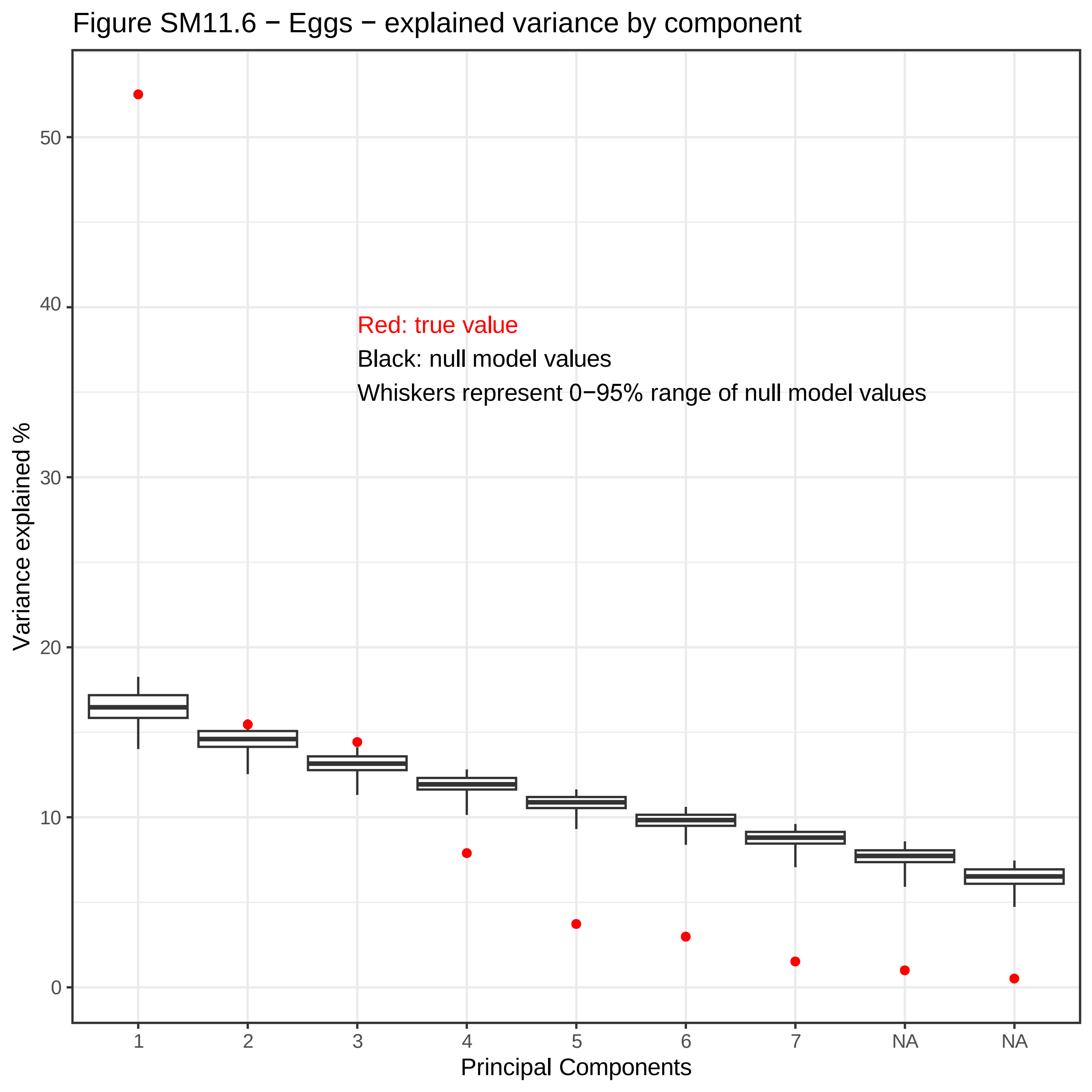

Figure SM11.6 − Eggs − explained variance by component
Red: true value
Black: null model values
Whiskers represent 0−95% range of null model values
Variance explained %
0
10
20
30
40
50
1
2
3
4
5
6
7
NA
NA
Principal Components

Figure SM11.7 − Animals pt − psi

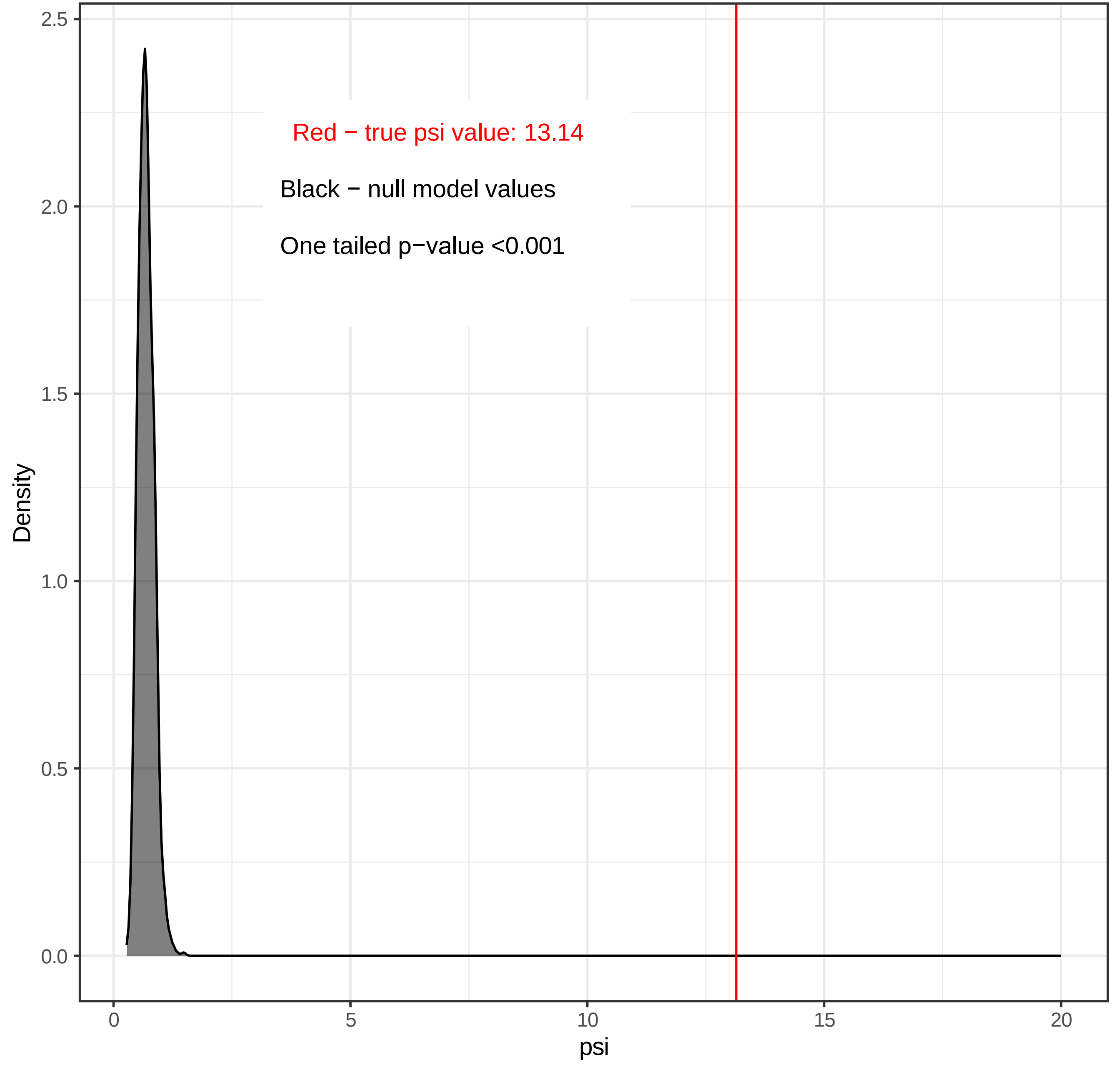

Figure SM11.8 − Animals pt − phi

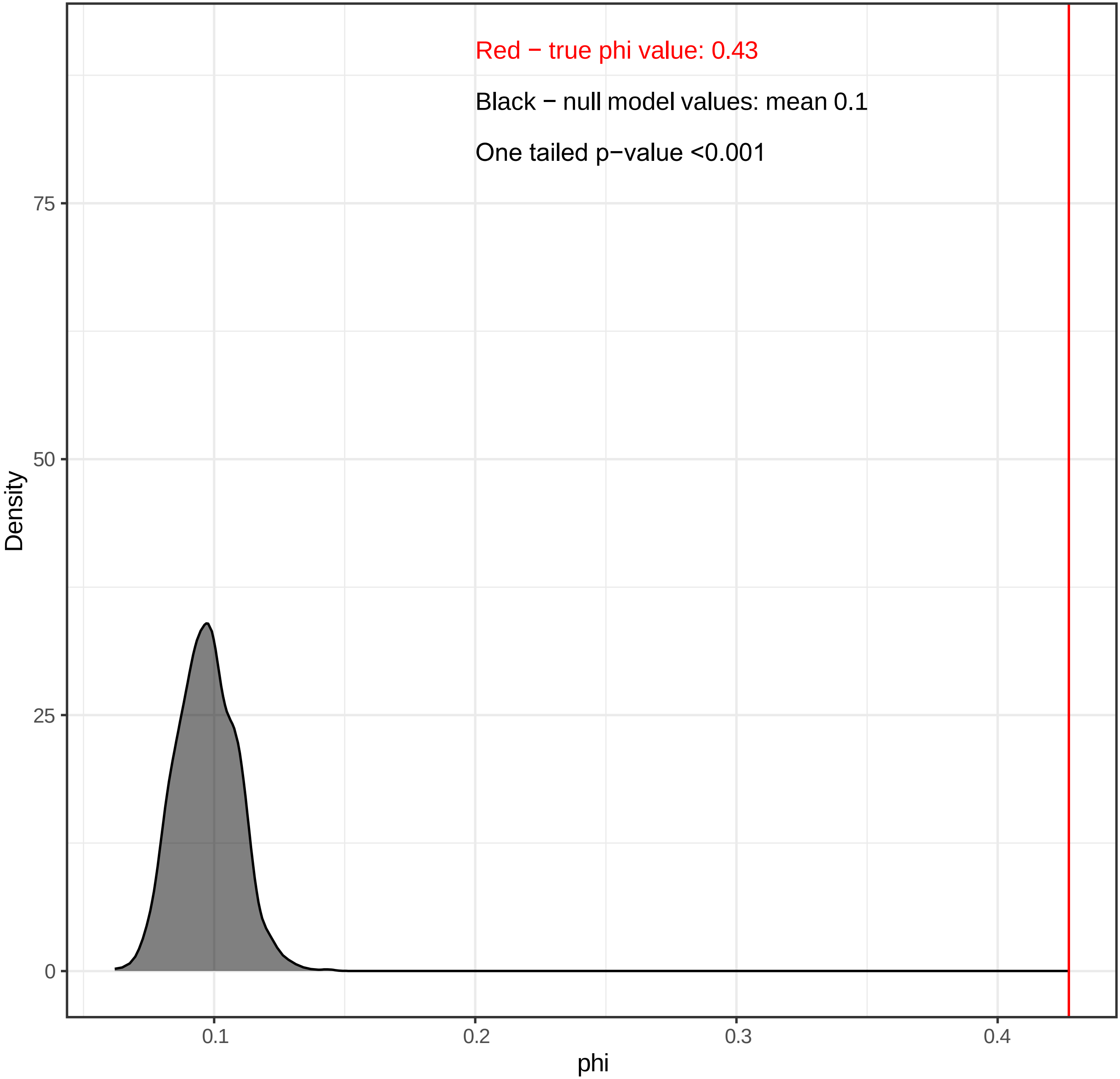

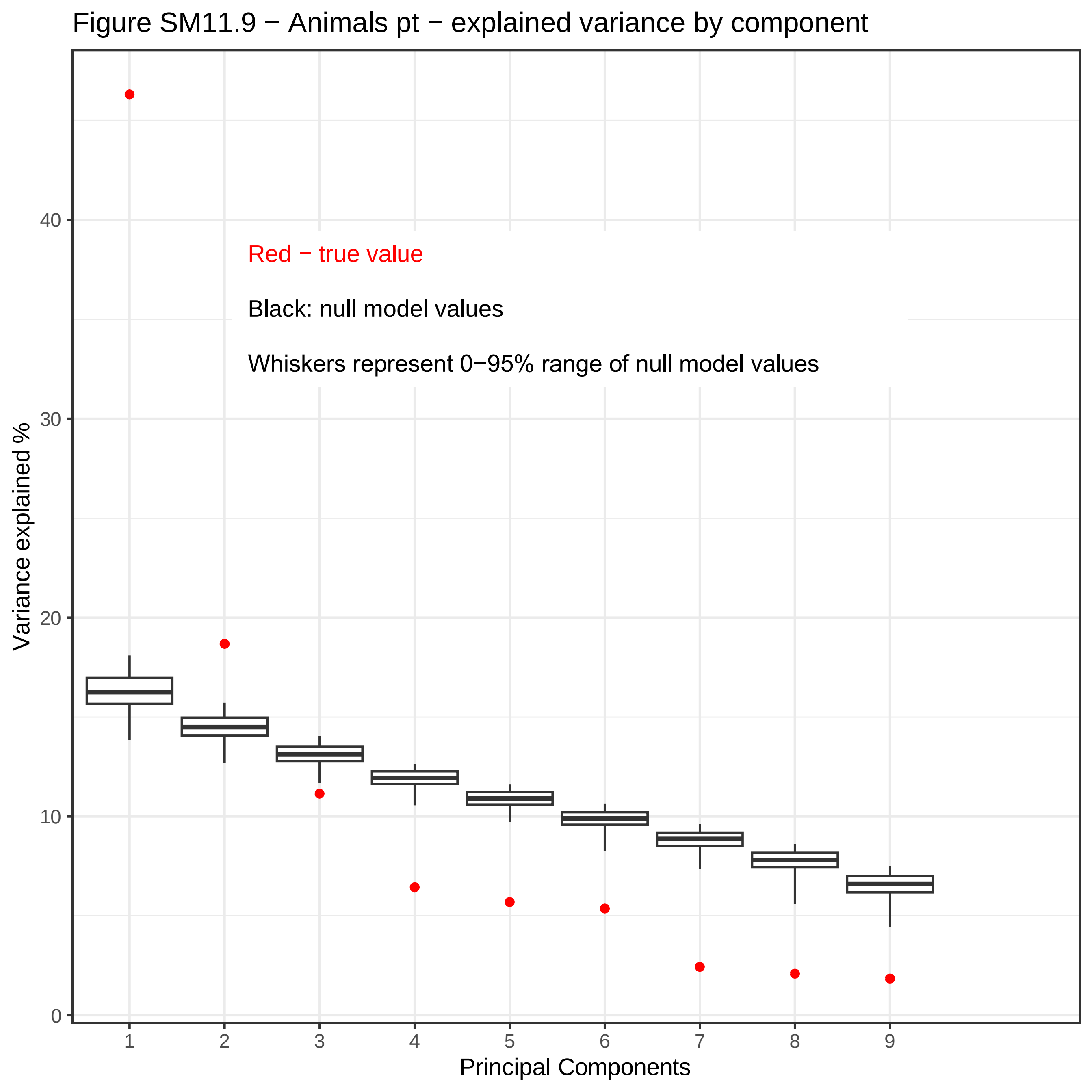
Figure SM11.9 − Animals pt − explained variance by component
Red − true value
Black: null model values
Whiskers represent 0−95% range of null model values
Variance explained %
40
30
20
10
0
1
2
3
4
5
6
7
8
9
Principal Components
